# The neural mechanism underpinning balance calibration between action inhibition and activation initiated by reward motivation

**DOI:** 10.1038/s41598-017-10539-z

**Published:** 2017-08-29

**Authors:** Hsin-Ju Lee, Fa-Hsuan Lin, Wen-Jui Kuo

**Affiliations:** 1 Institute of Neuroscience, National Yang-Ming University, Taipei, Taiwan; 2 Institute of Biomedical Engineering, National Taiwan University, Taipei, Taiwan; 3 Brain Research Center, National Yang-Ming University, Taipei, Taiwan

## Abstract

In everyday life, it is often the case that in some situations we are motivated and want not only to speed up our actions but also to avoid mistakes—for example, ballgames. How our brain works at that moment to resolve the situations and react properly has created an active research field. Previous findings indicated that maintaining a balance between withholding and executing an action are highly dynamic and involve many executive control processes. This fMRI study was set up to investigate how motivation affects these balancing processes. With manipulation of prospective rewards in a stop-signal task where both the proactive and reactive control were equally emphasized, our behavioral results replicated previous findings. The fMRI findings backed up the behavioral results. We found motivation effects in the anterior caudate and pre-SMA for action inhibition. The former works to register motivation status, the latter works to transform motivation into action inhibition control. Together with the results of connectivity analysis, our study also suggests a hierarchical relationship between functional roles of pre-SMA and right inferior frontal gyrus during action inhibition. While the pre-SMA acts to accommodate higher-order factors, such as motivation, for action control, the right inferior frontal cortex acts to participate in the execution of action inhibition. This study pinned down a neural mechanism that integrates reward motivation into action inhibition control.

## Introduction

It is often the case that in certain circumstances, we would like not only to speed up our actions or movements but also, meanwhile, to avoid mistakes—for example, while playing ballgames. During a game, a precise, dynamic balance between speeding up actions and avoiding mistakes at the same time are key to winning. However, the question about how the motivation to win adaptively adjust our action control and subsequent behavior remains to be answered. To this end, a well-established paradigm for action control, i.e., the stop-signal task^1^, was exploited. In a stop-signal task, the participants are asked to make a motor response as fast as they can to a response cue (a go-trial), and occasionally, before action execution, a stop-signal appears to call for stopping the intended action (a stop-trial). For stopping, the time interval between the response cue and the stop-signal—i.e., the stop-signal delay (SSD)—is critical. The shorter the interval, the easier the stopping. Based on the go response distribution and the unsuccessful stopping rate, we can calculate the processing time of a stop-signal (i.e., the stop-signal reaction time, SSRT) according to the racing model^1–3^. Application of this task together with fMRI and motivation manipulation is feasible and which allows us to evaluate the neural mechanism underpinning balance calibration between action inhibition and activation initiated by reward motivation.

Recently, there are a series of studies conducted to investigate how motivation induced by reward prospect modulates implementation of action inhibition, which include behavioral, fMRI, and electrophysiological studies^4–11^. Most of these studies demonstrated that promoting inhibition-reward prospect in a reactive manner could result in SSRT reduction^4–7, 10^. The finding is important because, without explicit instructions of proactive strategic preparation, it discloses the fact that reward prospect can facilitate processing of stop-signals that appear in a sudden and improve subsequent cancellation efficiency to an already-planned action in a very short period (~200 ms). The studies also indicate that although the stop-signals appear in a reactive manner, inhibition-reward prospect impacts not only on reactive control but also on proactive control to enhance reward-related inhibition processing and perceptual processing in general. Evidence for the former impact includes electrophysiological (the P3 effect)^4, 5^ and fMRI results^7^, which indicates that reward prospect regulates activities of the neural substrates, such as the pre-supplementary motor area (pre-SMA), for action inhibition. fMRI data also showed higher activity in ventral striatum when comparing brain activities associated with the rewarded stop-trials to those of the unrewarded stop-trials^7^. On the other hand, reward prospect proactively brought about top-down, attentional effects on early visual processing component (the N1 component), associated with the go stimuli of the stop-trials and the go-trials^4, 5, 8^. Altogether, these studies point out that, when motivated, regulation effects from reactive and proactive control intermingle with each other, and segregation of these two effects is practically intractable.

We know well that motivation affects behavior by driving us to approach rewards and to avoid punishment^12^. During the course, motivation either can facilitate our cognitive processing, and then speed up our actions by energizing the operation system^13, 14^ or it can cause rescheduling of priorities of various action goals^15^. It, therefore, leads to control over behavior through its linkage with various cognitive control systems, including working memory^16^, task-switching^17^, selective attention^9^, and action control^18, 19^. For neural substrates, in a recent review, the authors concluded that the ventral striatum and the medial prefrontal cortex are the two brain areas to host integration of cognitive control and motivation driven by reward^20^. However, please note that in those reviewed studies motivation strength and reward value closely coupled, which seriously complicates the interpretation of motivation effects. Recent evidence has indicated that motivation and reward value are dissociable in human striatum activities^21^. While activities in the caudate and putamen increase with motivation, nucleus accumbens activity increases with reward value. Therefore, because of the coupling between motivation and reward value, the conclusion aforementioned is not conclusive. The question about how the linkage between motivation and cognitive control for action works awaits further elucidation.

In this study, in order to avoid the involvement of intractable segregation between reactive and proactive control and the coupling between motivation strength and reward value, two critical experimental procedures were adopted. First, because of our major interest in the relationship between motivation and implementation of action inhibition, instead of segregation, we emphasized both reactive and proactive control at the same time by including a reward manipulation. To motivated the participants, they were told that they could earn an extra bonus (NT$2,000, about US$66.70) if their averaged action speed of the go-trials reached a given criteria that was established individually in a practice session. At the same time, they needed to be careful not to commit the stop-trial errors because there was a penalty—i.e., a loss of NT$40 (about US$1.30) per error from the possible bonus. By doing so, we created a situation where both the reactive and proactive control were encouraged altogether. The processing to maintain the balance between to go and to stop would be enhanced. Second, we employed a staircase-tracking procedure for the experimental task, by which we could fix the amount of final reward to all participants. The staircase-tracking procedure dynamically adjusts the SSD to maintain the response rate of stop-trials to be 50%, based on trial-by-trial stopping performance of the individuals^2^. The initial intent of the procedure was to improve the precision of SSRT estimates^2, 3, 22^. It also provides a feasible way for the current study to fix the amount of reward value for all participants. (See Methods for more details.) Based on this control for the stop-trial response rate (~50%), we could fix the amount of the reward and, therefore, avoid the coupling between motivation and reward value. Our design was in line with a previous study^11^, where the authors suggested that high task motivation can modulate proactive pre-stimulus inhibition of the go response. Their simulation results indicated, when task motivation was elevated, participants calibrate their timing in order to maximize the overall chance of success, including timely responses on go-trials as well as successfully inhibited stop-trials. The process of calibration, therefore, can result in a less positively skewed distribution of the go reaction time.

Neural substrates relevant to processing action inhibition have been depicted in many neuroimaging studies, and they comprise a fronto-basal-ganglia network including the right inferior frontal gyrus, pre-SMA, striatum, and the subthalamic nucleus (STN)^23, 24^. Many efforts have been devoted to investigating the functions of these areas and how they interact with each other as well^25–29^. For cortical areas of the network, research indicates that when there is a stopping process commencing from detection of a stop-signal, the right inferior frontal gyrus signals and commands the basal ganglia to stop the intended action^23^. Activity of this area negatively correlates with the individual’s SSRT^30–32^, which points to a direct, functional role of this area in controlling action inhibition. Another cortical area of the network, i.e., the pre-SMA, has functional and structural connections to the right inferior frontal gyrus^24, 28, 33^ and a structural connection to the basal ganglia input nuclei—i.e. the striatum^34^ and STN^24^. Transcranial direct current stimulation (tDCS) over the pre-SMA can affect stopping performance^35^. Instead of direct control, the pre-SMA seems to affect stopping performance in an indirect manner. For example, there is research^28^ showing that, by studying a medically intractable epileptic patient with intracranial grid coverage of the pre-SMA and right inferior frontal gyrus, stimulation of the pre-SMA evoked strong local field potentials within 30 ms in the right inferior gyrus. It also showed increased high gamma amplitude in both regions for preparation for stopping, with pre-SMA preceding right inferior frontal gyrus by ~750 ms. Moreover, it can represent motivation for a specific action in nonhuman primates and encode psychological states related to motivation, such as intention in humans^7, 36^. The results suggested a functional hierarchy of the pre-SMA and right inferior frontal gyrus in the stopping network. However, effective connectivity analysis from another study^26^ with a large fMRI data set suggested hierarchy of the two areas in the opposite direction. Therefore, although there are studies discussing their functional role and relationship in action inhibition^25–28^, the current status of how the pre-SMA and right inferior frontal gyrus interact to contribute to stopping an intended action, especially when motivation is highly engaged, is not yet clear, and it thereby inspires the current study.

In this study, for comparison, we included a control condition in which all its procedures were the same to those applied in the target situation except for the reward manipulation. The participants’ motivation level should be higher in the target situation and relatively lower in the control situation. Here, first, in light of previous computational model^11^, we specifically examined the skewness of RT distribution to test whether there is any proactive modulation effect on manipulation. Second, based on previous findings^7, 36, 37^ that the pre-SMA can represent motivation and encode psychological states related to motivation, we expected that pre-SMA activity would be influenced by motivation—i.e., the reward manipulation—and show responses in differential amplitudes to the stop-signals under different motivation contexts. Thirdly, based on the observations^23, 30–32^ that the right inferior frontal gyrus was more intrinsically, directly related to action inhibition ability, we also expected the right inferior frontal activities underlying successful stopping should negatively correlate with the SSRTs. By administering functional connectivity analysis, we planned to examine connectivity fluctuation between conditions, which could help to elucidate functional hierarchy of the pre-SMA and right inferior frontal gyrus.

## Material and Methods

### Participants

Eighteen healthy college students (mean age, 22; 10 males) took part in the experiment. All participants were neurologically intact, right-handed and had normal or corrected-to-normal vision. Informed consent was obtained before MR scanning from all participants, with the protocol approved by the Institutional Review Board of National Yang-Ming University. All experiments were performed in accordance with relevant guidelines and regulations. Participants received NT$500 (about US$16.68) for their participation. One data set was excluded from further analysis because the participant could not follow the instructions for completing two scanning sessions of the task.

### Stimuli and procedure

The stimuli were arranged and presented with the software E-Prime 2.0 (version 2.0.8.90, Psychology Software Tools Inc., Sharpsburg, PA, USA). Two geometric figures were used as response cues: a rhombus denoting a key press by the left index finger, and a pentagram denoting a key press by the right index finger. The mappings between response cues and effectors were counterbalanced across the participants. For stop-trials, a red cross (i.e., an X) was adopted to signal for stopping. All stimuli were centrally presented on the screen with a visual angle of about 1 degree. For any given trial, it started with a central fixation for a short period and then was replaced by a response cue—i.e. a rhombus or a pentagram—lasting for 1,000 ms for response. A stop-signal would superimpose on the response cue after a period of delay if it were arranged to be a stop-trial. Both response cues appeared equally as often in any trial type. The participants were instructed to respond to response cues as fast as they can and to stop the planned action as accurately as possible when a stop-signal appeared. Behavioral responses were recorded with an MR-compatible recording system.

There were two practice sessions before the fMRI scan. The first practice session included 60 go-trials, which allowed the participants to get familiar with the experimental setup and allowed us to collect their reaction times and set up an individual reaction-time baseline. The second session included 42 go-trials and 18 stop-trials, in which trials of the two types were randomly intermixed for practice. After practice, two fMRI scans were performed, one with and one without monetary manipulation. The sequential order of the two fMRI scans was counterbalanced across participants. Instruction of experimental condition was given at the beginning of each san. In each scan, there were 300 experimental trials, including 200 go-trials and 100 stop-trials. In order to increase efficiency and robustness for parameter estimation, there were 100 null trials included.

For stopping, we applied a one-up-one-down staircase-tracking procedure to adjust the SSDs of the stop-trials dynamically. The duration of the initial SSD was set at 200 ms. The SSD of the next stop-trial would be lengthened by 34 ms if the participants could withhold their response for the current stop-trial. If they could not, the next SSD would be shortened by 34 ms. In this way, we could control the response rate of the stop-trials at about 50%. For all participants, instructions were given at the beginning of the experiment. In the high-motivation situation, they were offered a bonus (NT$2,000, about US$66.70) if their averaged reaction time of the go-trials reached a given criterion established individually from the first practice session. In the meantime, the participants also needed to try to avoid error commitment for any stop-trials because there was a penalty for not being able to stop their action correctly—i.e., a loss of NT$40 (about US$1.30) per error from the possible bonus. In the control situation—i.e., the low-motivation situation—there was no bonus and penalty in company with their behavioral performance. However, the speed of the go-trials and accuracy of the stop-trials were still emphasized. In average, the payout was NT$28.2 (about US$0.94) in the high motivation condition.

### Image acquisition

Scanning was performed using a 3 T MRI (Tim Trio, Siemens, Erlangen, Germany) with a 32-channel head coil. Participants’ heads were immobilized with a vacuum-beam pad. A T2*-weighted gradient-echo echo planar imaging (EPI) sequence was used for fMRI scanning, with slice thickness = 3.4 mm, in-plane resolution (64 × 64) = 3.4 × 3.4 mm, and TR/TE/θ = 2,000 ms/30 ms/90°. Thirty-three axial slices were acquired to cover the whole brain. For each slice, 406 images were acquired in one run, lasting for about 14 minutes. The first two volumes of each run were discarded for signal equilibrium. Each participant’s anatomical image was acquired using a standard MPRAGE sequence (repetition time/echo time/inversion time [TR/TE/TI] = 2,530/3.03/1,100 ms, flip angle = 7°, partition thickness = 1 mm, image matrix = 256 × 256, 192 partitions, field of view = 22.4 cm × 22.4 cm) for within-subject registration purposes. The total duration of the experiment was approximately 45 minutes.

### Image processing and statistical analysis

Image data were preprocessed and analyzed using SPM software (http://www.fil.ion.ucl.ac.uk/spm/software/spm8). Functional images were corrected for slice timing and head motion, normalized to the MNI space, and spatially smoothed with an 8-mm FWHM Gaussian kernel. After pre-processing, the images were resliced into a resolution of 2 × 2 × 2 mm for each voxel. Statistical analysis was performed with the GLM approach and followed a two-level procedure. At the individual first-level analysis, the successful stop-trials (SST), the unsuccessful stop-trials (USST), the correct go-trials (GO), the trials of error, and the six movement parameters under the low- and high-motivation situations were included as regressors for estimation. Effects of interest were examined with the contrasts constructed by linear combination of beta estimates of the regressors. Contrast images of the SST, the USST, and the GO under high- and low-motivation situations were derived and entered into the second-level analysis for group results. At the second level, several comparisons were conducted to examine motivation effects on the SST, the USST, and the GO. For the GO, a paired-t test was used. For the SST and USST, a 2 × 2 flexible ANOVA analysis was administered. Unless stated otherwise, activations were thresholded at voxel level p < 0.005 and corrected at cluster level with an *FWE* p < 0.05. Therefore, inferences of the imaging results should base on the level of the entire cluster. All coordinates were reported in the MNI coordinate space.

### ROI and functional connectivity analysis

To avoid the non-independence error for ROI analysis, a leave-one-subject-out (LOSO) procedure was applied to define the ROIs^38^. In the procedure, the ROIs used for each participant were defined by the group results from the rest of the participants. For example, here, we had seventeen participants. Coordinates of the ROIs used for the first participant were defined by the group results of the rest (another sixteen) participants. Therefore, we iteratively performed the same procedure for all participants to build their ROIs for further analysis. In this study, there were three ROIs of interest to be defined: one in the medial prefrontal cortex (i.e., the pre-SMA), one in the right inferior frontal gyrus, and one in the caudate structure. In the results, we found significantly higher activities in the pre-SMA and caudate for the SST in the high motivation condition (see the Results section). With our specific interest and its given characteristics in action inhibition, the right inferior frontal gyrus was selected as one of the ROIs. Its center coordinate was defined by looking for the intersection point of the stopping main effects (i.e., the SST and USST under both conditions) in the right inferior frontal gyrus. Regarding the pre-SMA and right inferior gyrus ROIs, a spherical volume with a radius of 6 mm was applied to cover the target areas. For the caudate ROI, because of bilateral activation, a spherical volume with a radius of 4 mm was applied to the left and right caudate areas, and centered at the coordinate of the peak activity, respectively. Beta estimates of the regressors (the SST and USST) were extracted from the three ROIs. A simple linear regression analysis was performed to test if activities of the pre-SMA and right inferior frontal ROIs correlated with individuals’ SSRT from the two motivation situations, respectively. A repeated ANOVA was performed to examine activities of the pre-SMA and caudate ROIs with motivation and stopping success as two within-subject factors.

Functional connectivity by seeding from the pre-SMA ROI was examined using the psychophysiological interaction (PPI) method^39^. In this study, we employed a generalized form of context-dependent PPIs (gPPIs) that has the flexibility to accommodate more than two conditions in the same PPI model^40^. We obtained estimates of neural activities from the seeding region by deconvolving the extracted BOLD signals. The psychological vectors used in the PPI analysis include regressors of the SST, the USST, and the GO under high- and low-motivation situations. The physiological, psychological and PPI vectors were then convolved with the canonical hemodynamic response function and entered as regressors at a first-level general linear model. For the effects of our interest, two PPI contrasts were created: 1) the SST versus the USST during the high-motivation situation, and 2) the SST versus the USST during the low-motivation situation. The contrast images from all participants were examined using a paired t-test at the second level. The results were thresholded at voxel level p < 0.005 and corrected at cluster level with an *FWE* p < 0.05.

## Results

### Behavioral data

To examine the motivation effect over action control, error rates and reaction times of the GO, reaction times of the USST, and SSRTs under the high- and low-motivation situations were calculated (see Table 1 for the details). Application of the staircase-tracking procedure for SSD adjustment yielded an action inhibition rate of about 50%. The SSRT was derived using the integration method^2, 3^, by which the mean SSD is subtracted from the RT of the n^th^ go-trial, in which n is calculated by multiplying the total number of the correct go-trial with the responding rate of the stop-trials (about 50%).

**Table 1:** Descriptive statistics (mean and standard deviation) for Go and Stop trials across motivation states.

	Motivation state	
	Low	High
***GO***		
Error rate (%)	3.21 (2.36)	1.59 (1.53)
RT (ms)	571 (96)	559 (83)
Sknewness of RT distribution	0.34 (0.25)	0.25 (0.24)
***STOP***		
Signal-respond RT (ms)	523 (76)	507 (68)
Inhibition rate (%)	52.41 (1.84)	52.35 (1.97)
SSD (ms)	341 (96)	326 (92)
SSRT (ms)	216 (26)	222 (24)

Note: SSRT = stop-signal reaction time.

For the GO, most errors were omission errors, and the error rate was higher in the low-motivation situation than in the high-motivation situation (3.21% vs. 1.59%; t(16) = 3.541, *p* = 0.001). Although the two averaged RTs of the two situations were not significantly different (571 ± (s.d.) 96 ms for low motivation, 559 ± 83 ms for high motivation; t(16) = 1.001, *p* = 0.166), the RT distribution of the GO from the high-motivation situation is less positively skewed (low-motivation skewness = 0.341, high-motivation skewness = 0.245; t(16) = 2.148, *p* = 0.046).

For the stop-trials, RTs of the USST from the two situations were significantly shorter than those of the GO from the two situations (low-motivation: 523 ± 76 ms vs. 571 ± 96 ms; t(16) = 7.684, p < 0.001; high –motivation: 507 ± 68 ms vs. 559 ± 83 ms; t(16) = 10.084, *p* < 0.001). The inhibition rates were 52.35% and 52.41%, and the estimated SSRTs were 216 ms and 222 ms for the low- and high-motivation situations, respectively. The difference between the two estimated SSRTs is not significant (t(16) = 0.946, *p* = 0.179).

### fMRI data

#### The main effects

**Figure 1:**
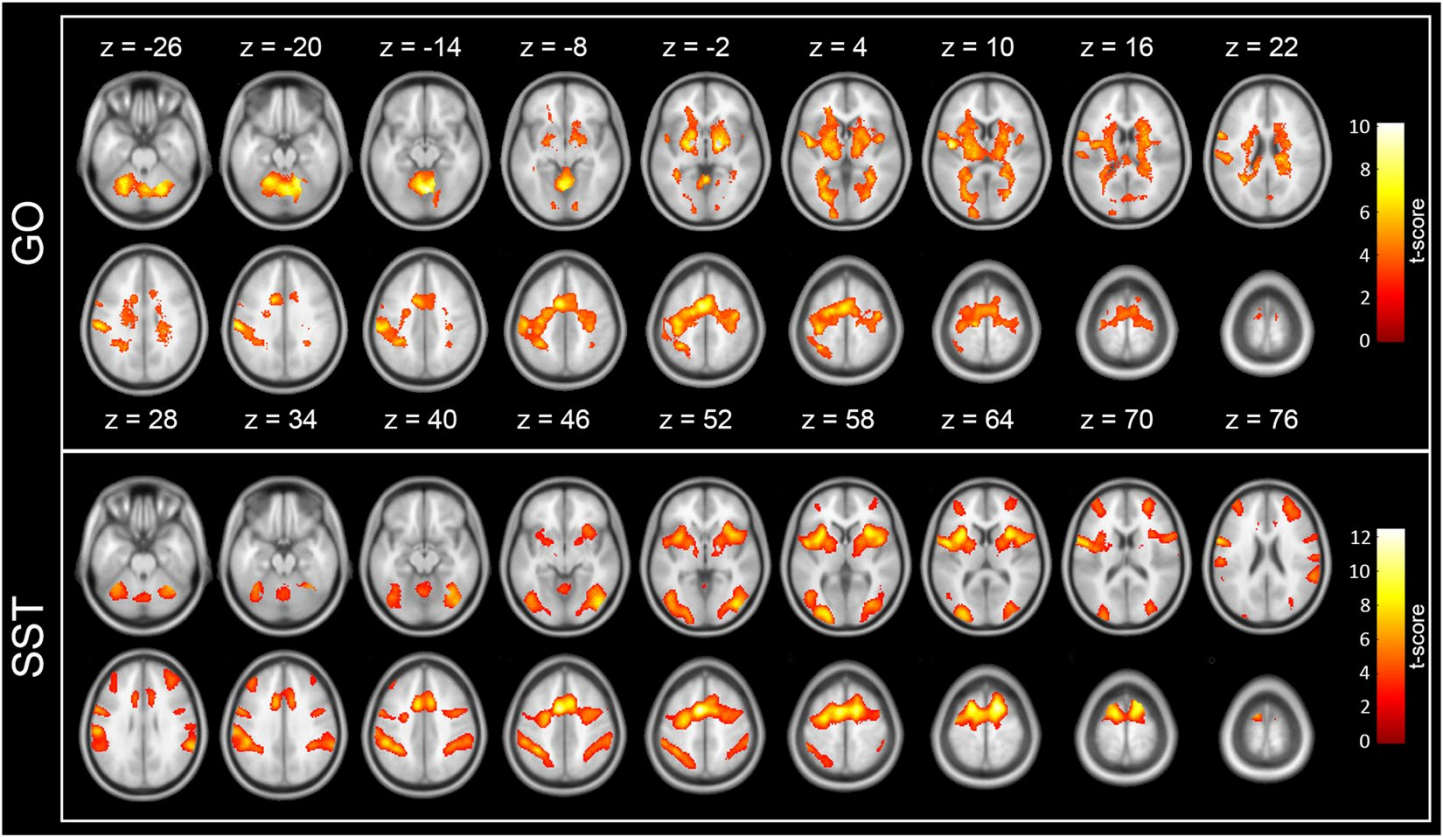
Main effects for the GO and stop-trials in the low-motivation situation. (the upper part) The GO resulted in activities in the precentral gyrus, premotor cortex, supplementary motor areas (SMA), superior temporal cortex, right superior parietal cortex, cerebellum, as well as subcortical regions such as the caudate, putamen, globus pallidum, and thalamus. (the lower part) Both the SST and USST caused activities in the precentral gyrus, medial frontal gyrus, anterior cingulate cortex, middle prefrontal gyrus, inferior frontal gyrus, inferior parietal cortex, middle occipital gyrus, cerebellum, and basal ganglia.

The GO resulted in activities in the precentral gyrus, premotor cortex, supplementary motor areas (SMA), superior temporal cortex, right superior parietal cortex, cerebellum, as well as subcortical regions such as the caudate, putamen, globus pallidum, and thalamus (the upper part of Fig. 1). Both the SST and USST caused activities in the precentral gyrus, medial frontal gyrus, anterior cingulate cortex, middle prefrontal gyrus, inferior frontal gyrus, inferior parietal cortex, middle occipital gyrus, cerebellum, and basal ganglia (the lower part of Fig. 1).

To examine whether motivation had exerted its effect over the GO, the SST, and the USST, we compared effects of the three trial types from the high- to the low-motivation situations and presented the results as follows.

#### Motivation modulation over the GO

**Figure 2:**
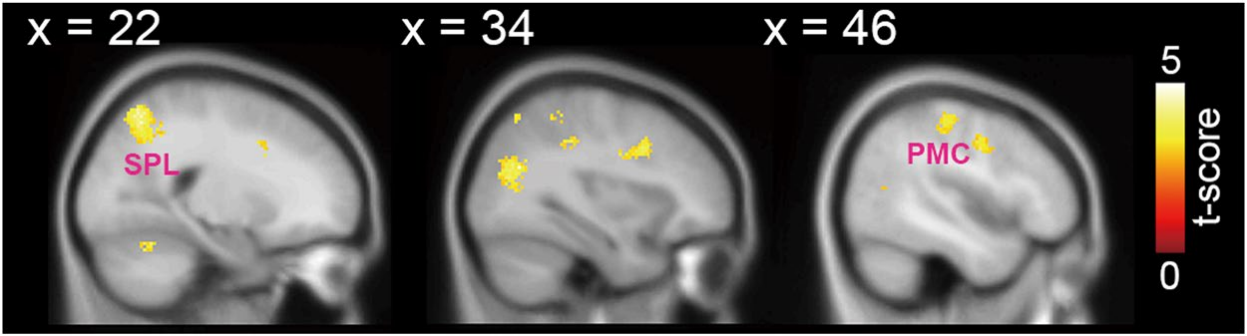
Motivation modulation over the GO. Compared to the low-motivation situation, the right PM and right SPL, parts of the fronto-parietal networks, were found to show greater activation during the GO in the high-motivation situation. Greater activities in the fronto-parietal network suggest that the participants were engaged and paid more attention to the response cues when they expected to be rewarded.

Compared to the low-motivation situation, the GO activities from the high-motivation situation were greater in the right premotor, right superior parietal lobule, postcentral gyrus, and bilateral cerebellum (see Fig. 2 and Table 2). Greater activities in the fronto-parietal network suggested that the participants were more engaged and paid more attention to the response cues.

**Table 2:** Summary of significant activations associated with the motivation modulation.

Anatomical region	Hemi-sphere	Cluster size	Brodmann Area	MNI coordinate			t-value
				x	y	z	
***GO***							
Inferior Frontal Gyrus	R	522	9	62	14	26	3.56
Middle Frontal Gyrus			6	32	10	44	3.84
Precentral Gyrus			6	56	8	34	3.3
				56	4	40	3.17
				42	−6	38	4.41
Precuneus	R	1,190	7	24	−54	44	4.25
Superior parietal lobule			7	18	−62	60	4.33
				26	−66	56	4.97
				32	−68	56	3.6
Cerebellum	L/R	600		4	−52	−8	4.17
				−6	−54	−6	4.22
				−8	−68	−26	4.39
				2	−68	−16	4.21
				−4	−72	−30	4.23
***SST***							
Pre-SMA	L/R	726	8	−6	42	38	3.00
				−2	34	44	3.22
				0	28	42	3.16
				2	26	48	3.59
				8	26	48	3.34
Caudate	L/R	627		8	6	10	3.65
				10	2	10	3.93
				−8	0	14	3.85
Thalamus				8	−8	2	3.36
				−12	−26	14	3.34

Note: The data are thresholded at p < 0.005 (uncorrected) for voxels and p < 0.05 (FWE-corrected) for clusters.

#### Motivation modulation over the SST and the USST

**Figure 3:**
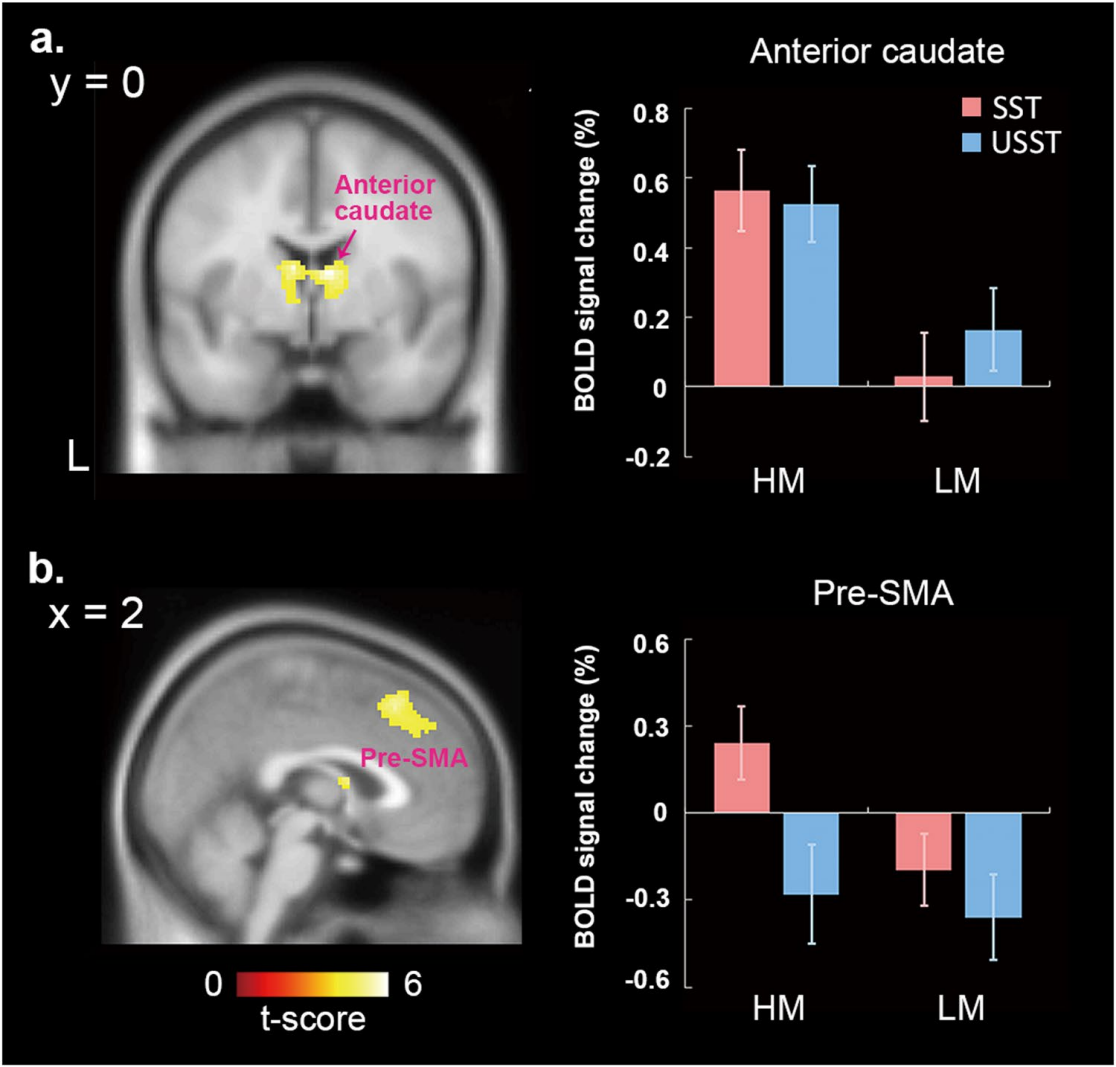
Motivation effects over the stopping processing. Greater activities in the anterior caudate (**a**) and pre-SMA (**b**) were found during SSTs for the high-motivation situation. To inspect activities of the anterior caudate and pre-SMA in more detail, parameter estimates of the two areas were extracted and examined by within-factors ANOVA analysis. The anterior caudate (**a**) only shows the motivation main effect, indicating activities of this area were greater when motivation was high, regardless of inhibition success. For the pre-SMA (**b**), brain activity of the SST was higher than that of the USST in the high-motivation situation, but no difference was seen in the low-motivation situation. (HM = high motivation; LM = low motivation).

Motivation modulation over the SST and the USST were separately examined. For the former, greater activities in the anterior caudate and pre-SMA were found for the high-motivation situation (see Fig. 3a,b). However, for the latter, there was no significant motivation effect. In order to inspect activities of the caudate and pre-SMA in more details to test our hypotheses, parameter estimates of the two areas were extracted and further submitted to an ROI analysis.

#### ROI and functional connectivity analysis

**Figure 4:**
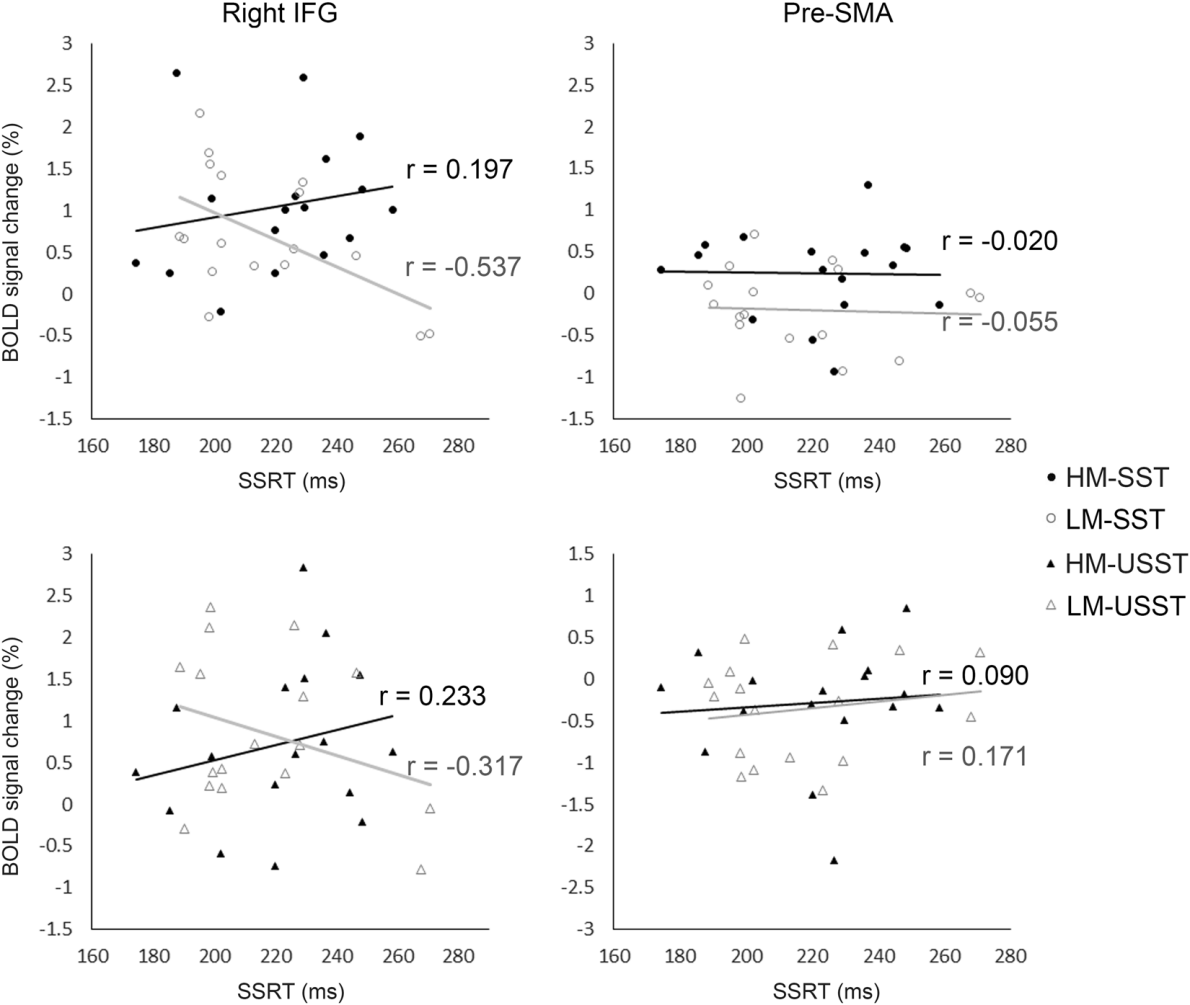
Correlation between the SSRTs and BOLD signals extracted from the right inferior frontal gyrus (left) and pre-SMA (right). In the right inferior frontal gyrus, a negative correlation between the SSRT and its activity of the SST showed in the low-motivation situation (r = –0.504, t = –2.260, p = 0.039), but not in the high-motivation situation (r = 0.322, t = 1.320, p = 0.207). It suggested that the right inferior frontal gyrus works to direct action inhibition in regular situations. However, when motivated by reward prospect, it results in decorrelation between activity in the right inferior frontal gyrus and the SSRT. Neither the USST nor the pre-SMA, no correlation displayed with the SSRTs. (HM = high motivation; LM = low motivation).

Figure 4 shows the results of simple regression analysis that was planned for the right inferior frontal gyrus and the pre-SMA. In the right inferior frontal gyrus, there was a negative correlation between SSRT and its activity of the SST in the low-motivation situation (r = −0.537, t = −2.463, *p* = 0.026), but not in the high-motivation situation (r = 0.197, t = 0.780, *p* = 0.447). In the pre-SMA, no correlation between SSRT and its activity of the SST was revealed in either situation (low-motivation: r = −0.055, t = −0.213, p = 0.834; high-motivation: r = −0.020, t = −0.077, p = 0.939). For the USST, neither rIFG (low-motivation: r = −0.317, t = −1.294, p = 0.215; high-motivation: r = 0.233, t = 0.927, p = 0.447) nor pre-SMA (low-motivation: r = 0.171, t = 0.671, p = 0.513; high-motivation: r = 0.090, t = 0.348, p = 0.732) showed the correlation between SSRTs and its activities.

For the anterior caudate, motivation (high and low) and inhibition success (the SST and the USST) were included as two within-subject factors for an ANOVA analysis. There was only a main effect revealed—i.e., the motivation (F(1, 16) = 13.259, *p* = 0.002). The activities of this area were greater when motivation was high, regardless of inhibition success (see Fig. 3a).

In respect of the pre-SMA, motivation (high and low) and inhibition success (the SST and the USST) were included for two variables for analysis as well. Both the main effects of motivation and inhibition success (motivation: F(1, 16) = 6.458, *p* = 0.022; inhibition success: F(1, 16) = 8.877, *p* = 0.009) and the interaction effect (F(1, 16) = 5.157, *p* = 0.037) were significant. Brain activity of the SST was higher than that of the USST in the high-motivation situation (simple main effect using paired-t test with a Bonferroni adjustment: t(16) = 3.891, *p* = 0.002), but no difference was seen in the low-motivation situation (see Fig. 3b).

**Figure 5:**
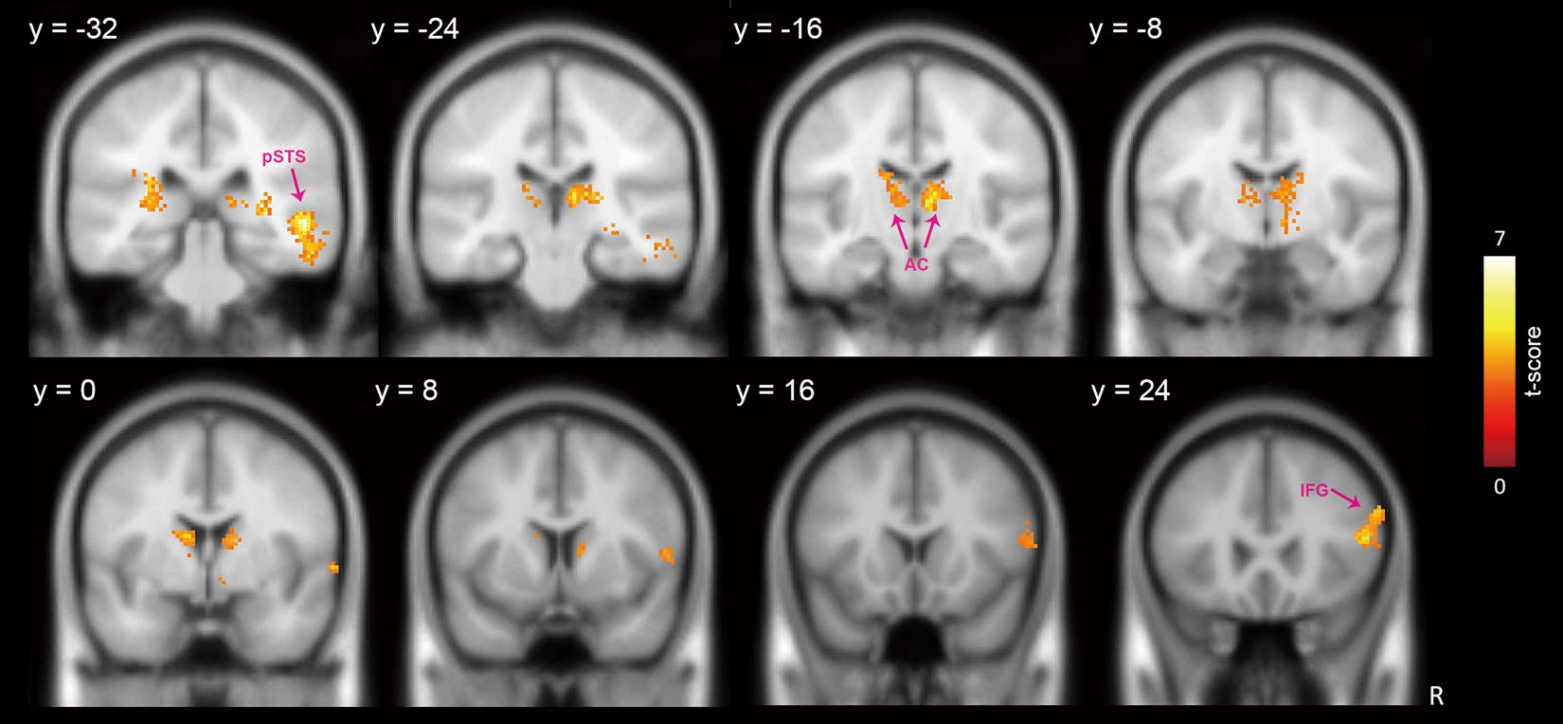
Brain areas showing strengthened coupling with the pre-SMA between motivation levels. In comparison to the task when participants were not motivated, the right pIFG, anterior caudate and pSTS showed strengthened coupling with the pre-SMA in the psychological contrast of SST versus USST when participants were motivated. Activations were thresholded at voxel level *p* < 0.005 and corrected at cluster level with a FWE *p* < 0.05. (pIFG = posterior inferior frontal gyrus; AC = anterior caudate; pSTS = posterior superior temporal sulcus.

Given that the pre-SMA is a part of the stopping network and plays an important role in action control^25–28^, functional connectivity of the pre-SMA with other brain areas was examined using a generalized context-dependent psychological interaction analysis method^40^. When applying the psychological contrast of SST versus USST, the results indicate that positive coupling between pre-SMA activity and activities of the right posterior IFG (BA45), anterior caudate and right posterior superior temporal sulcus (pSTS) was stronger in the high-motivation situation than in the low-motivation situation (see Fig. 5 and Table 3).

**Table 3:** Pre-SMA seeded PPI results. In contrast to the low-motivation situation, brain regions showed significant functional connectivity with pre-SMA on the psychological contrast of SST versus USST in the high-motivation situation.

Anatomical region	Hemi-sphere	Cluster size	Brodmann area	MNI coordinate			t-value
				x	y	z	
Inferior frontal gyrus	R	304	45	54	28	8	3.13
			44	58	20	12	3.39
				60	12	8	2.94
Middle frontal gyrus			9	58	26	28	4.07
Caudate	L	483		−12	0	16	4.28
				−14	−14	22	3.78
Thalamus				−8	−20	8	4.26
				−10	−20	12	4.23
				−20	−28	16	3.92
Caudate	R	619		12	4	12	4.20
				14	−4	16	4.67
Thalamus				8	−12	2	4.25
				8	−20	8	5.43
				14	−20	12	4.21
Posterior superior temporal sulcus	R	405	22	52	−32	−2	6.90

Note: The data are thresholded at p < 0.005 (uncorrected) for voxels and p < 0.05 (FWE-corrected) for clusters.

## Discussion

The aim of this study was to investigate the linkage between motivation and action inhibition control by incorporating a reward manipulation with a stop-signal task and to examine its neural underpinnings by using fMRI. We learned from previous studies that segregation between reactive and proactive control is intractable and coupling between motivation strength and reward value can be dissociable. In the current study, first, instead of segregation, we emphasized reactive and proactive control at the same time during the task. Secondly, we fixed the amount of final monetary reward to all participants to avoid this coupling by applying a staircase-tracking procedure. Our results could help elaborate how reward motivation regulates the implementation of action inhibition.

### Behavioral data

With the emphasis on both proactive and reactive control during the task, we found two motivational effects. First, missing rate of the GO was lower in the high-motivation situation, suggesting that the participants were motivated and became more alert to the response cues. Second, the GO RT distribution of the high motivation condition was less positively skewed, which corroborated the expectation suggested by a previous computational model^11^. The less positive skewness means that the participants apply a strategy to better balance their wobbling between fast going and accurate stopping. In the high motivation condition, both attempts of the participants to respond quickly and to stop accurately should compete much harder. The participants would develop a strategy to balance fast response and accurate stopping to maximize reward availability, through calibrating their timing for response. Balancing the two competing forces, therefore, result in a shift of the GO RT distribution to become more symmetric, but not reduction in the GO RT and SSRT. On the other hand, motivation may also exert its influence at the pre-stimulus stage^11^ or proactively promote the efficiency of subsequent perceptual processing in general^4, 5, 8^, instead of direct impact on the stopping itself.

We are aware that there have been studies showing that SSRT can be either shortened or lengthened by manipulations to bias the participants’ response strategy^4–7, 9, 10^. The SSRT becomes shorter when the SST is rewarded. On the other hand, the SSRT becomes longer when the GO is going to be rewarded. In other words, relative to a proactive relevance as shown in ours and Liddle *et al*.’s^11^ study, when there is a reward selectively offered to a particular response, it results in a response bias and a subsequent reactive SSRT change. However, when a more thorough inspection was administered, although there were SSRT changes, both effects of reactive and proactive control evolved and mingled along the course^4–7, 9, 10^. Segregation of the two effects is intractable. Therefore, in our study, we paved a way in a more natural sense to examine how reward motivation regulates implementation of action inhibition in a stop-signal task. Although the newly established balance was invisible in behavioral data, fMRI data could help delineate how we calibrated the balance from its neural aspect.

### fMRI data

The fMRI results brought us fruitful information to scrutinize effects of reward motivation over action inhibition and relevant processes. There were two main motivation effects, i.e., the reactive inhibition and proactive facilitation. We examined the former by looking at motivation modulation over the SST and the latter by taking a close look at motivation modulation over the GO. Results of functional connectivity provided grounding information to address functional hierarchy of the pre-SMA and right inferior frontal gyrus in inhibition control.

#### Motivation modulation over the GO

For the GO, the fronto-parietal networks revealed greater activation in the high-motivation situation. Since the GO was randomly presented and occupied a large proportion of the experimental trials (66.67%), this finding suggested that the reward motivation caused higher engagement of cognitive control of the participants. It lays a neural foundation for the observations that the participants are more alert to the response cues and, therefore, commit fewer missing errors during the task. This finding is consistent with a recent study that was conducted to describe the function of the dorsal fronto-parietal networks while performing goal-directed actions^41^. In our case, in order to better balance rapid response and accurate stopping, the dorsal fronto-parietal networks for controlling and monitoring the participants’ behaviors were more engaged. This finding soundly agrees with the inference of setting a balance to maximize the reward prospect^11^. It also concurs with the electrophysiological findings that indicate the involvement of attentional/proactive control in a stop-rewarded situation^5, 8^.

#### Motivation modulation over the SST

We found stopping-related activities in a wide range of brain areas distributed in the frontal and parietal cortices, and subcortical structures, which replicated a number of previous findings about action inhibition^7, 9, 24, 26, 28, 30, 42–44^. However, only the pre-SMA and anterior caudate were disclosed as showing a motivation effect. Regarding the anterior caudate, its activity is shown to be sensitive to motivation, but indifferent to inhibition success (see Fig. 3a). Higher motivation resulted in higher activity for both the SST and the USST, suggesting that this area registers motivation status and subsequently forwards this message to the motor/action control systems. There is evidence supporting this idea. In nonhuman primates, the caudate incorporates reward anticipation in oculomotor control^45–48^. Human functional imaging studies reveal that the caudate activities can represent reward information in goal-directed actions^49–51^. Therefore, the function manifested here for the anterior caudate is to register motivation status, which is also consistent with observations of functional segregation in the human striatum^21^.

The pre-SMA is another area surviving from the contrast as the anterior caudate does, suggesting a functional relevance between these two areas. This relevance confirms previous observations that the pre-SMA has structural and functional connections to the anterior caudate^34, 50, 52, 53^. Together with co-activation of the anterior caudate, we propose that the anterior caudate prospectively transforms its motivation effect into influence over the pre-SMA. Indeed, there is supportive evidence for this proposal. Neurophysiological recording of rhesus monkeys shows that a class of pre-SMA neurons can represent motivation for a specific action^37^. In humans, pre-SMA activity is found to be able to encode psychological states related to motivation, such as intention^36^. In this current study (see Fig. 3b), the pre-SMA activity was higher for the SST than for the USST only when motivation was high. It points to a possibility that, when motivated (receiving reward message from anterior caudate), the pre-SMA will strengthen its communication with downstream action-inhibition circuits to improve stopping efficiency to an intended action. Activity fluctuations of this area, thus, give a substantial influence on inhibition success.

Regression analysis to test correlations between the individuals’ SSRT and both their pre-SMA and right inferior frontal activities provides information from another perspective to examine how the stopping processes proceed. The results only showed a significant negative correlation between the SSRT and right inferior frontal activity in the low-motivation situation (see Fig. 4). This finding replicated the results of several previous studies^30–32^, revealing that the right inferior frontal gyrus works to direct action inhibition. It is interesting and noteworthy that this correlation becomes weaker when the participants were motivated, implying that the motivation here‒i.e., winning more money‒will perturb action inhibition directed by the right inferior frontal control. This perturbation can be more easily realized and explained when taking into account the pre-SMA seeded PPI results.

#### Connectivity among areas

The PPI analysis indicated that the pre-SMA shows a stronger coupling with the right inferior frontal cortex in the high-motivation situation. That is, motivation gives its influence to action inhibition by modulating the connectivity between the pre-SMA and the right inferior frontal gyrus. Taken together with the regression results, the indication is that the inferior frontal control over action inhibition holds in regular situations. However, when motivated by reward prospect, in order to establish a new balance^11^, the pre-SMA seems to inform the right inferior frontal gyrus and reshape its control over action inhibition, which results in decorrelation between its activity and the SSRT. Our findings, in general, are consistent with a recent study that describes the indirect role of the pre-SMA in stopping action^27^ and other studies that show structural and functional connections between the pre-SMA and the prefrontal cortex^24, 28, 33, 36, 52, 54^.

The PPI results indicated the pre-SMA activity also coupled with activities of the pSTS and the anterior caudate. The finding of the anterior caudate replicates the relevance demonstrated in the contrast of the SSTs under high- and low-motivation situations. However, the findings on the pSTS, although unexpected, are understandable. We know that the pSTS is crucial for understanding and coding our actions^55–59^. In this study, our participants might have been more concerned with and, therefore, more aware of their action status when there was a prospective reward expected. This finding suggests the existence of this awareness. However, further investigation is needed to confirm this conjecture.

## Conclusion

Understanding the neural underpinnings of the linkage between motivation and action control has attracted much attention and invited many studies for research. In this fMRI study, rather than segregation, we emphasized reactive and proactive control at the same time to pave a more natural way to inspect motivational effects on action inhibition. To do this, we adopted a stop-signal task together with manipulation of prospective rewards. With avoidance to the coupling between motivation strength and reward value, our behavioral data support the concept that motivation introduces establishment of a new balance between fast response and accurate stopping^11^. The fMRI findings back up the behavioral results. Establishment of a new balance draws on involvement of the fronto-parietal networks. For processing stop-signals, the motivation effect takes place in the anterior caudate and pre-SMA. The former works to register motivation status, whereas the latter works to transform motivation into action inhibition control. The results of our correlation and connectivity analysis suggest a hierarchical relationship between functional roles of the pre-SMA and right inferior frontal gyrus during action inhibition. While the pre-SMA acts to accommodate higher-order factors—such as motivation—for action control, the right inferior frontal cortex acts to participate in execution of action inhibition directly.
